# Hepatocellular Carcinoma Surveillance and Survival in a Contemporary Asia-Pacific Cohort

**DOI:** 10.1001/jamanetworkopen.2025.20294

**Published:** 2025-07-11

**Authors:** Ryan Yanzhe Lim, Benjamin Koh, Cheng Han Ng, Anand V. Kulkarni, Ken Liu, Karn Wijarnpreecha, Beom Kyung Kim, Mark D. Muthiah, Sung Won Lee, Ming-Hua Zheng, Takumi Kawaguchi, Hirokazu Takahashi, Daniel Q. Huang

**Affiliations:** 1 Yong Loo Lin School of Medicine, National University of Singapore, Singapore; 2 Division of Gastroenterology and Hepatology, Department of Medicine, National University Hospital, Singapore, Singapore; 3 Division of Gastroenterology, Department of Medicine, Kurume University School of Medicine, Kurume, Japan; 4 Department of Hepatology, Asian Institute of Gastroenterology, Hyderabad, India; 5 A.W. Morrow Gastroenterology and Liver Centre, Australian Liver Transplant Unit, Royal Prince Alfred Hospital, Sydney, New South Wales, Australia; 6 Sydney Medical School, University of Sydney, Sydney, New South Wales, Australia; 7 Division of Gastroenterology and Hepatology, Department of Medicine, University of Arizona College of Medicine, Phoenix; 8 Department of Internal Medicine, Banner University Medical Center, Phoenix, Arizona; 9 Department of Internal Medicine, Yonsei University College of Medicine, Seoul, Republic of Korea; 10 Yonsei Liver Center, Severance Hospital, Seoul, Republic of Korea; 11 National University Centre for Organ Transplantation, National University Health System, Singapore, Singapore; 12 Division of Hepatology, Department of Internal Medicine, Catholic University of Korea, Seoul, Republic of Korea; 13 MAFLD Research Center, Department of Hepatology, The First Affiliated Hospital of Wenzhou Medical University, Wenzhou, China; 14 Liver Center, Saga University Hospital, Saga, Japan; 15 Division of Metabolism and Endocrinology, Department of Medicine, Faculty of Medicine, Saga University, Saga, Japan

## Abstract

**Importance:**

The etiologies of hepatocellular carcinoma (HCC) are changing. It is unclear whether the benefit of surveillance for people with HCC remains consistent given the changing etiologies of the disease.

**Objective:**

To evaluate the association of HCC surveillance with the survival rates of a large contemporary cohort of people with HCC.

**Design, Setting, and Participants:**

This was a retrospective cohort study that included participants with HCC between January 2008 and August 2023. Participants with HCC were identified from 5 international sites in Singapore, Japan, South Korea, and Australia. A total of 1185 adult participants were included in this cohort study. All included participants had available information regarding the presence or absence of surveillance. Data were analyzed from June 26, 2024, to March 6, 2025.

**Main Outcomes and Measures:**

The primary outcome was overall survival rates with HCC surveillance vs no surveillance. Multivariable restricted mean survival time (RMST) analyses with lead-time bias adjustments were conducted to assess overall survival.

**Results:**

Of 1185 participants with HCC, 921 (77.7%) were male, the mean (SD) age of the participants was 67.6 (10.7) years, and the mean (SD) body mass index was 25.7 (5.3). RMSTs were consistently higher for participants who underwent HCC surveillance (n = 975) (RMST difference at 1 year, 0.10 years [95% CI, 0.06-0.15 years]; *P* < .001; RMST difference at 3 years, 0.50 years [95% CI, 0.32-0.68 years]; *P* < .001; RMST difference at 5 years, 0.96 years [95% CI, 0.64-1.29 years]; *P* < .001) compared with participants who did not undergo surveillance (n = 210). RMSTs remained higher among participants with hepatitis B (HBV) or hepatitis C (HCV) who underwent surveillance compared with those with no surveillance across all follow-up periods. Among participants with metabolic dysfunction–associated steatotic liver disease (MASLD) and alcohol-associated liver disease, there were no statistically significant differences in RMSTs in the first 3 years of follow-up between HCC surveillance and no surveillance.

**Conclusions and Relevance:**

This cohort study of 1185 participants with HCC found that HCC surveillance was associated with improved survival. This survival benefit was more prominent for people with HBV-associated and HCV-associated HCC. The survival benefit of surveillance was less consistent for people with MASLD-associated or alcohol-associated HCC, which may have been related to the relatively modest sample size in the nonviral groups.

## Key Points

**Question:**

What is the association of surveillance with the survival of people with hepatocellular carcinoma (HCC), considering the changing etiologies of HCC?

**Findings:**

In this cohort study of 1185 participants with HCC, the restricted mean survival times (RMSTs) were consistently higher for participants who underwent HCC surveillance. RMSTs remained higher among participants with hepatitis B virus and hepatitis C virus under surveillance but not metabolic dysfunction–associated steatotic liver disease (MASLD) or alcohol-associated liver disease.

**Meaning:**

These findings suggest that HCC surveillance was associated with improved survival rates, with a consistent survival benefit for participants with hepatitis B virus–associated or hepatitis C virus–associated HCC; the survival benefit of surveillance was less consistent for participants with MASLD-associated or alcohol-associated HCC, which may have been related to the relatively modest sample size in the nonviral groups.

## Teaser

This cohort study evaluates the association of hepatocellular carcinoma (HCC) surveillance with the survival of a large contemporary cohort of people with HCC identified from 5 international sites in Singapore, Japan, South Korea, and Australia.

## Introduction

Hepatocellular carcinoma (HCC) is the third leading cause of cancer death worldwide and is associated with poor long-term survival.^1^ The 5-year survival rate of HCC in general is less than 20%.^2^ Tumor stage at the time of diagnosis is a major determinant of prognosis; 5-year survival rates exceed 55% among patients with early-stage disease, but median survival among those with advanced tumors is less than 2 years.^2,3,4,5,6,7^ By contrast, surveillance for HCC is associated with early detection, curative treatment receipt, and improved survival rates.^8^ Guidelines from major societies, including the American Association for the Study of Liver Diseases, the European Association for the Study of the Liver, and the Asia-Pacific Association for the Study of the Liver recommend semiannual ultrasonographic scans for people with cirrhosis and for selected individuals with chronic hepatitis B.

The etiology of HCC has changed substantially in the recent decade.^9,10,11^ The burden of HCC associated with MASLD and alcohol-associated liver disease is increasing, whereas death rates of HCC associated with hepatitis B virus and hepatitis C virus are decreasing.^12,13,14^ Emerging data suggest that HCC surveillance may have poorer surveillance test performance in people with MASLD.^15,16,17,18^ A meta-analysis identified only 2 studies that examined the association between HCC surveillance in people with MASLD and survival, with 1 study reporting no difference in survival between those who underwent surveillance and those who did not.^8,19^ It is unclear whether HCC surveillance is consistently associated with improved survival rates given the changing etiologies of liver disease. Therefore, we examined the association between HCC surveillance and survival in a large contemporary Asian-Pacific cohort of participants with HCC.

## Methods

### Study Design

This is a retrospective, multicenter, Asian-Pacific cohort study of consecutive adults with HCC between January 2008 to August 2023. Participants were recruited from 5 international tertiary health care institutes from Singapore (National University Hospital), Japan (Saga University Hospital and Kurume University Hospital), South Korea (Severance Hospital), and Australia (Royal Prince Alfred Hospital). Each institute’s institutional review board approved this study and waived informed consent because the study involved deidentified data in accordance with 45 CFR 46. This study was performed per the ethical principles of the Declaration of Helsinki and Istanbul, and applicable regulatory requirements. All clinical, laboratory, and imaging data were collected from a review of individual patient medical records using a standardized case report form and standardized definitions for data collection. Deidentified data were transmitted to and analyzed at the National University of Singapore. This study followed the Strengthening the Reporting of Observational Studies in Epidemiology (STROBE) reporting guideline for cohort studies.

### Study Population and Definitions

Consecutive adult participants (≥18 years of age) were included in the study. All included participants had available information regarding the presence or absence of surveillance. Participants without data for surveillance, date of HCC diagnosis, date of death or the date of last contact, and erroneously recorded date of diagnosis or date of death or last contact were excluded. HCC surveillance was defined as ultrasonography, computed tomography, or magnetic resonance imaging performed at any frequency for asymptomatic participants for the specific intent of HCC screening within the preceding 18 months. Imaging performed for symptoms or incidentally detected HCC on imaging not performed for screening intent or for the confirmation of HCC diagnosis was not considered HCC surveillance. Follow-up cross-sectional imaging for an isolated elevated α-fetoprotein that was not ordered in tandem with an imaging test for the specific purpose of surveillance was not considered HCC surveillance. HCC was diagnosed following the criteria outlined in the American Association for the Study of Liver Diseases guidelines, including imaging with Liver Imaging Reporting and Data System category 5 lesions and/or histological confirmation.^20^ MASLD was defined as evidence of hepatic steatosis and the exclusion of significant alcohol consumption (≥14 drinks per week for men or ≥7 drinks per week for women) and other alternative causes of hepatic steatosis, such as viral hepatitis.^21^ Participants who were previously classified as having cryptogenic cirrhosis with obesity (defined as either body mass index [BMI; calculated as weight in kilograms divided by height in meters squared] ≥27.5 for Asian participants or a BMI ≥30.0 for White participants) and/or type 2 diabetes were considered to have MASLD and were included, based on the recommendations of a multistakeholder workgroup.^22^ The index date was the date of initial HCC diagnosis to the date of death or the date of last contact. Cirrhosis was defined based on histology or imaging evidence of cirrhosis via ultrasonography, computed tomography, or magnetic resonance imaging, including left and caudate lobe enlargement with right lobe shrinkage, gallbladder fossa expansion, blunt liver edge, irregular or nodular liver surface, the presence of portal hypertension, along with associated features including reversal of portal flow, splenomegaly, splenic vein dilation, and ascites.^23,24^ Surgical resection, ablation, and liver transplant were considered curative treatments for HCC. Milan criteria for liver transplant was defined as having a single lesion smaller than 5 cm or up to 3 lesions each smaller than 3 cm, no extrahepatic manifestations, and no evidence of gross vascular invasion.^25^ Other baseline characteristics collected included aspartate aminotransferase, alanine aminotransferase, and maximal tumor diameter. The primary objective of this study was overall survival with HCC surveillance vs no surveillance.

### Statistical Analysis

Baseline characteristics were compared using the χ^2^ test for categorical variables and the *t* test and Wilcoxon rank sum test for parametric and nonparametric continuous variables, respectively. Univariable and multivariable restricted mean survival time (RMST) analyses were used to evaluate the differences in overall survival between people with HCC who underwent surveillance and those who did not undergo surveillance to address nonproportionality, adjusted for age, sex, race and ethnicity (comparing Asian patients with White patients), type 2 diabetes, BMI, and Child-Pugh-Turcotte classification.^26,27^ The RMST model provides a robust estimation of survival in the presence of proportionality violation and estimates the difference in mean survival times (RMST difference). RMST differences can be calculated at specific cutoff points. As opposed to a hazard ratio, for which a value of less than 1.00 is associated with a better outcome, a larger RMST value represents longer event-free survival time and equates to improved clinical outcomes. Multivariable analyses were conducted using the analysis of covariance–type method, a regression-based method for estimating RMST differences (eMethods 1 in Supplement 1).^28,29^ Lead-time adjustments were performed using the parametric method proposed by Duffy et al,^30^ assuming a mean sojourn time of 5 months as previously described (eMethods 2 in Supplement 1).^31^ Participants with multiple etiologies for liver disease, such as HCV with alcohol-associated liver disease, were included in the analysis under each of their contributing etiologies. Sensitivity analyses were conducted using mean sojourn times of 4 and 6 months for lead-time adjustments, after excluding participants with multiple etiologies for liver disease, and after excluding participants with less than 6 months of follow-up.^32^ Crude and adjusted RMST differences were estimated at 1, 2, 3, 4, and 5 years of follow-up. Data were analyzed from June 26, 2024, to March 6, 2025, with R, version 4.4.0 (R Project for Statistical Computing) using the survRM2 package, and a 2-sided *P* < .05 was considered statistically significant.

## Results

### Characteristics of the Study Cohort

A total of 1185 HCC participants (921 male participants [77.7%]) were included in this study, of whom 975 underwent HCC surveillance and 210 did not undergo surveillance (eFigure in Supplement 1). The baseline characteristics are summarized in Table 1. The number of participants with hepatitis B virus (HBV)–associated, hepatitis C virus (HCV)–associated, MASLD-associated, and alcohol-associated liver disease was 422 (35.6%), 395 (33.3%), 178 (15.0%), and 168 (14.2%), respectively. The proportion of patients with HBV-, HCV-, MASLD-, and alcohol-associated liver disease who fulfilled guideline recommendations for HCC surveillance was 81.6%, 89.6%, 87.9%, and 78.8%, respectively. A total of 464 patients (39.2%) died during the study period. The mean (SD) age was 67.6 (10.7) years, and the mean (SD) BMI was 25.7 (5.3); 836 participants with HCC (70.5%) were Asian, 678 (57.4%) had cirrhosis, and 414 (34.9%) had type 2 diabetes. α-Fetoprotein levels (8.5 ng/mL vs 20.9 ng/mL [to convert to micrograms per liter, multiply by 1.0]; *P* < .001), and maximal tumor diameter (2.4 cm vs 3.8 cm; *P* < .001) were lower for participants who underwent HCC surveillance compared with those who did not undergo surveillance. The proportion with Barcelona Clinic Liver Cancer stage 0/A (428 [77.8%] vs 52 [38.2%]; *P* < .001), within Milan criteria (152 [82.2%] vs 26 [44.8%]; *P* < .001), who received curative treatments (597 [61.2%] vs 99 [47.1%]; *P* < .001) was higher for participants who received HCC surveillance compared with those who did not receive surveillance. The mean (SD) follow-up was 3.8 (3.0) years, whereas the median follow-up was 3.3 years (IQR, 1.2-5.8 years).

**Table 1.** Baseline Characteristics of People With HCC

Characteristic	Participants, No. (%)			*P* value
	Overall cohort (n = 1185)	HCC surveillance (n = 975)	No HCC surveillance (n = 210)	
Age, mean (SD), y	67.6 (10.7)	67.9 (10.6)	66.3 (11.2)	.05
Sex				
Male	921 (77.7)	754 (77.3)	167 (79.5)	.49
Female	264 (22.3)	221 (22.7)	43 (20.5)	
Ethnicity				
Asian	836 (70.5)	682 (69.9)	154 (73.3)	.33
White	349 (29.5)	293 (30.1)	56 (26.7)	
BMI	25.7 (5.3)	25.7 (5.3)	25.5 (5.0)	.51
Cirrhosis	678 (57.4)	584 (60.0)	94 (45.2)	<.001
Type 2 diabetes	414 (34.9)	341 (35.0)	73 (34.8)	.95
History of alcohol use	327 (35.2)	268 (34.3)	59 (39.9)	.19
History of tobacco use	262 (28.3)	199 (25.6)	63 (42.9)	<.001
Platelets, 10^9^/L	115 (56-184)	103 (48-170.5)	173 (108.8-246)	<.001
INR	1.10 (1.01-1.24)	1.10 (1.01-1.25)	1.10 (1.00-1.21)	.07
AST, U/L	44 (29-77)	43 (28.5-73)	50 (32-106.8)	<.001
ALT, U/L	35 (23-63)	35 (23-60)	39 (25-73)	.04
AFP, ng/mL	9.3 (3.7-93.5)	8.5 (3.6-72.7)	20.9 (4.4-960.3)	<.001
Child-Pugh-Turcotte classification				
A	511 (44.8)	409 (43.5)	102 (51.3)	.12
B	555 (48.7)	471 (50.1)	84 (42.2)	
C	74 (6.5)	61 (6.5)	13 (6.5)	
Maximum tumor diameter, cm	2.6 (1.8-4.5)	2.4 (1.7-3.8)	3.8 (2.6-7.5)	<.001
BCLC Stage 0/A	480 (70.0)	428 (77.8)	52 (38.2)	<.001
Received curative treatment	696 (58.7)	597 (61.2)	99 (47.1)	<.001
Within Milan criteria	178 (73.3)	152 (82.2)	26 (44.8)	<.001
Mean (SD) follow-up time, y	3.8 (3.0)	4.0 (3.0)	2.5 (2.6)	<.001
Median (IQR) follow-up time, y	3.3 (1.2-5.8)	3.6 (1.6-6.0)	1.5 (0.3-4.6)	<.001

Abbreviations: AFP, α-fetoprotein; ALT, alanine aminotransferase; AST, aspartate aminotransferase; BCLC, Barcelona Clinic Liver Cancer; BMI, body mass index (calculated as weight in kilograms divided by height in meters squared); HCC, hepatocellular carcinoma; INR, international normalized ratio.

SI conversion factors: To convert AFP to micrograms per liter, multiply by 1.0; and ALT and AST to microkatals per liter, multiply by 0.0167.

### Overall Survival of the Entire Cohort

**Figure 1.**
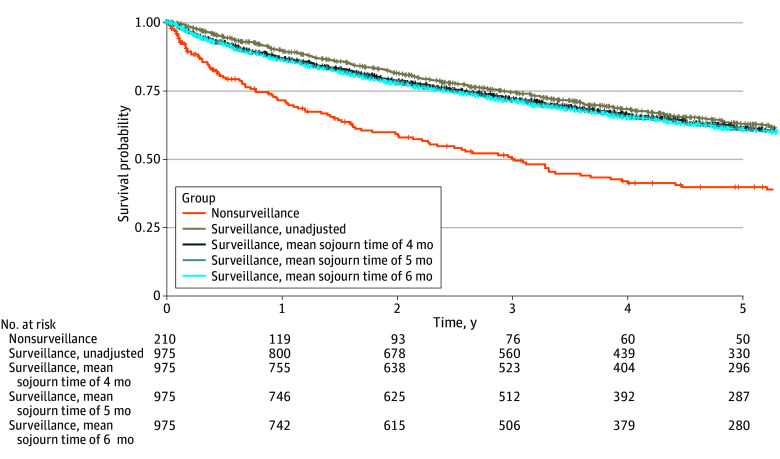
Kaplan-Meier Curves of Overall Study Cohort Comparing Participants Who Underwent Hepatocellular Carcinoma Surveillance With Those Who Did Not

In univariable analyses, the RMST was consistently higher in the surveillance group compared with the nonsurveillance group across all 5 years of follow-up (Figure 1; eTable 1 in Supplement 1). After adjustment for lead-time bias (5-month mean sojourn time), HCC surveillance remained associated with significantly higher RMST time compared with no HCC surveillance.

HCC surveillance remained associated with significantly higher RMST after adjusting for lead time (5-month mean sojourn time), age, sex, ethnicity, type 2 diabetes, BMI, and Child-Pugh-Turcotte classification (RMST difference at 1 year, 0.10 years [95% CI, 0.06-0.15 years]; *P* < .001; RMST difference at 3 years, 0.50 years [95% CI, 0.32-0.68 years]; *P* < .001; RMST difference at 5 years, 0.96 years [95% CI, 0.64-1.29 years]; *P* < .001) (Table 2). Sensitivity analyses with mean sojourn times of 4 and 6 months indicated consistent findings.

**Table 2.** Multivariable Analysis of People With Hepatocellular Carcinoma Who Underwent Surveillance Compared With Those Who Did Not Undergo Surveillance

Variable	Restricted mean survival time, y^a^									
	At 1 y		At 2 y		At 3 y		At 4 y		At 5 y	
	Difference (95% CI)	*P* value	Difference (95% CI)	*P* value	Difference (95% CI)	*P* value	Difference (95% CI)	*P* value	Difference (95% CI)	*P* value
Crude (unadjusted)	0.13 (0.08-0.17)	<.001	0.35 (0.24-0.46)	<.001	0.58 (0.40-0.76)	<.001	0.84 (0.59-1.09)	<.001	1.10 (0.78-1.42)	<.001
Adjusted for lead time										
Mean sojourn time of 4 mo	0.11 (0.06-0.15)	<.001	0.30 (0.19-0.41)	<.001	0.51 (0.33-0.69)	<.001	0.75 (0.50-1.00)	<.001	0.99 (0.66-1.31)	<.001
Mean sojourn time of 5 mo	0.10 (0.06-0.15)	<.001	0.29 (0.18-0.40)	<.001	0.50 (0.32-0.68)	<.001	0.73 (0.48-0.98)	<.001	0.96 (0.64-1.29)	<.001
Mean sojourn time of 6 mo	0.10 (0.05-0.15)	<.001	0.28 (0.17-0.39)	<.001	0.48 (0.30-0.67)	<.001	0.71 (0.46-0.96)	<.001	0.94 (0.61-1.26)	<.001

^a^ Adjusted for sex, age, ethnicity, type 2 diabetes, body mass index, and Child-Pugh-Turcotte classification.

### Overall Survival, Stratified by Etiology of Liver Disease

**Figure 2.**
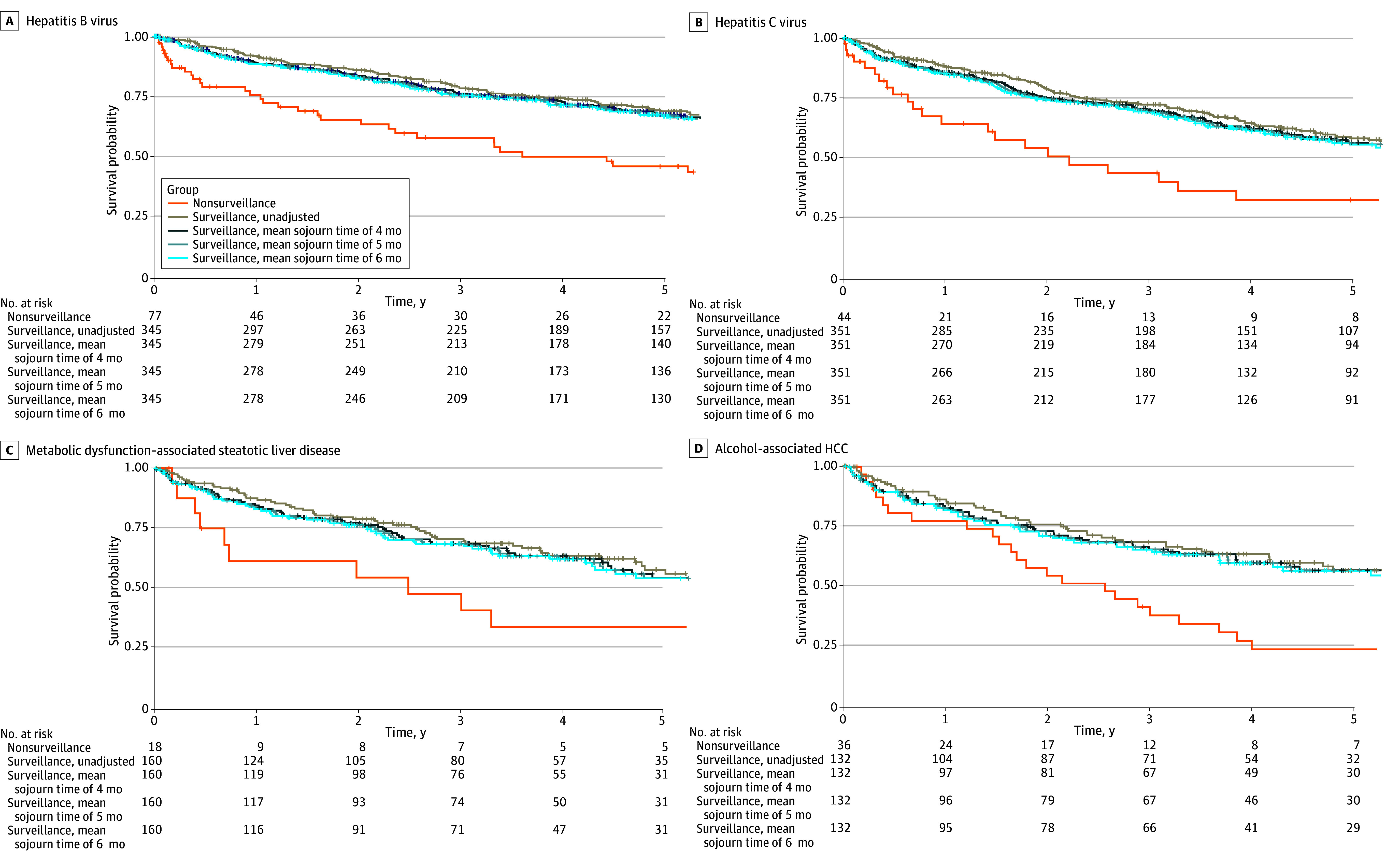
Kaplan-Meier Curves of Participants With Hepatitis B Virus–Associated Hepatocellular Carcinoma (HCC), Hepatitis C Virus–Associated HCC, Metabolic Dysfunction–Associated Steatotic Liver Disease–Associated HCC, and Alcohol-Associated HCC

In univariable analysis, surveillance was associated with consistently higher RMSTs among participants with HBV (RMST difference at 1 year, 0.11 years [95% CI, 0.03-0.19 years]; *P* = .008; RMST difference at 3 years, 0.47 years [95% CI, 0.18-0.77 years]; *P* = .002; RMST difference at 5 years, 0.90 years [95% CI, 0.37-1.43 years]; *P* = .001) and HCV (RMST difference at 1 year, 0.12 years [95% CI, 0.01-0.22 years]; *P* = .03; RMST difference at 3 years, 0.58 years [95% CI, 0.18-0.97 years]; *P* = .005; RMST difference at 5 years, 1.12 years [95% CI, 0.43-1.81 years]; *P* = .001) compared with no surveillance, including after adjustment for lead-time bias (Figure 2A and B; eTables 2-5 in Supplement 1). However, there were no statistically significant differences in RMSTs in the first 3 years between HCC surveillance vs no surveillance, among people with MASLD (Figure 2C), nor in the first 2 years, among people with alcohol-associated liver disease (Figure 2D).

After adjustment for lead time, age, sex, ethnicity, type 2 diabetes, BMI, and Child-Pugh-Turcotte classification, surveillance remained associated with consistently higher RMSTs among participants with HBV and HCV compared with no surveillance (Table 3). By contrast, there were no statistically significant differences in RMSTs in the first 3 years between HCC surveillance and no surveillance among people with MASLD and those with alcohol-associated liver disease after adjustment for lead time, age, sex, ethnicity, type 2 diabetes, BMI, and Child-Pugh-Turcotte classification. There was a statistically significant increase in RMST in the HCC surveillance group in the fourth and fifth years for both MASLD (RMST difference at 4 years, 1.05 years [95% CI, 0.09-2.01 years]; *P* = .03; RMST difference at 5 years, 1.34 years [95% CI, 0.07-2.62 years]; *P* = .04) and alcohol-associated liver disease (RMST difference at 4 years, 0.68 years [95% CI, 0.10-1.25 years]; *P* = .02; RMST difference at 5 years, 1.02 years [95% CI, 0.26-1.78 years]; *P* = .008). After excluding participants with multiple etiologies of liver disease, surveillance remained associated with consistently higher RMSTs among participants with HBV, whereas there were no statistically significant differences in RMSTs in the first year among people with HCV, the first 3 years among people with alcohol-associated liver disease, and all 5 years among people with MASLD (eTable 6 in Supplement 1). Exclusion of participants with less than 6 months of follow-up showed statistically insignificant differences in RMSTs between HCC surveillance and no surveillance across all etiologies of liver disease (eTable 7 in Supplement 1); this may be associated with the removal of patients who did not receive surveillance and passed away within 6 months of HCC diagnosis.

**Table 3.** Multivariable Analysis of People With Hepatocellular Carcinoma Who Underwent Surveillance Compared With Those Who Did Not Undergo Surveillance, Stratified by Etiology

Etiology	Restricted mean survival time, y^a^									
	At 1 y		At 2 y		At 3 y		At 4 y		At 5 y	
	Difference (95% CI)	*P* value	Difference (95% CI)	*P* value	Difference (95% CI)	*P* value	Difference (95% CI)	*P* value	Difference (95% CI)	*P* value
**Hepatitis B virus**										
Lead time unadjusted	0.13 (0.05 to 0.20)	.001	0.32 (0.14 to 0.49)	<.001	0.53 (0.25 to 0.81)	<.001	0.74 (0.34 to 1.13)	<.001	0.97 (0.46 to 1.48)	<.001
Lead time adjusted										
Mean sojourn time of 4 mo	0.11 (0.03 to 0.18)	.005	0.28 (0.11 to 0.45)	.002	0.47 (0.19 to 0.75)	.001	0.67 (0.27 to 1.06)	.001	0.89 (0.37 to 1.41)	.001
Mean sojourn time of 5 mo	0.10 (0.03 to 0.18)	.006	0.27 (0.10 to 0.45)	.002	0.46 (0.17 to 0.74)	.002	0.65 (0.25 to 1.04)	.001	0.87 (0.35 to 1.39)	.001
Mean sojourn time of 6 mo	0.10 (0.03 to 0.18)	.007	0.27 (0.09 to 0.44)	.003	0.44 (0.16 to 0.73)	.002	0.63 (0.24 to 1.03)	.002	0.85 (0.33 to 1.37)	.001
**Hepatitis C virus**										
Lead time unadjusted	0.15 (0.04 to 0.26)	.009	0.42 (0.16 to 0.69)	.002	0.70 (0.28 to 1.12)	.001	0.98 (0.42 to 1.55)	.001	1.21 (0.49 to 1.94)	.001
Lead time adjusted										
Mean sojourn time of 4 mo	0.13 (0.01 to 0.24)	.03	0.37 (0.10 to 0.64)	.007	0.61 (0.19 to 1.03)	.004	0.88 (0.31 to 1.45)	.003	1.07 (0.33 to 1.81)	.005
Mean sojourn time of 5 mo	0.13 (0.01 to 0.24)	.03	0.36 (0.09 to 0.62)	.009	0.59 (0.18 to 1.01)	.005	0.86 (0.29 to 1.43)	.003	1.04 (0.30 to 1.79)	.006
Mean sojourn time of 6 mo	0.12 (0.01 to 0.24)	.04	0.35 (0.08 to 0.61)	.01	0.58 (0.16 to 1.00)	.007	0.82 (0.25 to 1.39)	.005	1.01 (0.27 to 1.76)	.008
**Metabolic dysfunction–associated steatotic liver disease**										
Lead time unadjusted	0.16 (0.00 to 0.31)	.05	0.40 (−0.01 to 0.81)	.06	0.69 (0.02 to 1.36)	.04	1.12 (0.19 to 2.04)	.02	1.49 (0.23 to 2.74)	.02
Lead time adjusted										
Mean sojourn time of 4 mo	0.13 (−0.03 to 0.29)	.10	0.36 (−0.06 to 0.78)	.10	0.64 (−0.05 to 1.33)	.07	1.04 (0.11 to 1.98)	.03	1.37 (0.10 to 2.64)	.04
Mean sojourn time of 5 mo	0.13 (−0.03 to 0.29)	.11	0.35 (−0.07 to 0.78)	.10	0.64 (−0.06 to 1.33)	.07	1.05 (0.09 to 2.01)	.03	1.34 (0.07 to 2.62)	.04
Mean sojourn time of 6 mo	0.13 (−0.03 to 0.29)	.12	0.35 (−0.07 to 0.78)	.10	0.64 (−0.06 to 1.33)	.07	1.04 (0.07 to 2.01)	.04	1.32 (0.04 to 2.60)	.04
**Alcohol-associated liver disease**										
Lead time unadjusted	0.09 (−0.01 to 0.20)	.08	0.25 (0.00 to 0.50)	.05	0.49 (0.08 to 0.91)	.02	0.80 (0.24 to 1.36)	.005	1.18 (0.44 to 1.91)	.002
Lead time adjusted										
Mean sojourn time of 4 mo	0.07 (−0.03 to 0.18)	.18	0.20 (−0.07 to 0.46)	.14	0.41 (−0.02 to 0.83)	.06	0.70 (0.12 to 1.27)	.02	1.04 (0.29 to 1.80)	.007
Mean sojourn time of 5 mo	0.07 (−0.04 to 0.18)	.19	0.19 (−0.08 to 0.45)	.17	0.39 (−0.03 to 0.82)	.07	0.68 (0.10 to 1.25)	.02	1.02 (0.26 to 1.78)	.008
Mean sojourn time of 6 mo	0.07 (−0.04 to 0.18)	.21	0.17 (−0.09 to 0.44)	.20	0.38 (−0.05 to 0.80)	.08	0.66 (0.08 to 1.25)	.03	0.99 (0.23 to 1.76)	.01

^a^ Adjusted for sex, age, ethnicity, type 2 diabetes, body mass index, and Child-Pugh-Turcotte classification.

## Discussion

In this large, multinational study of 1185 participants with HCC, we determined that HCC surveillance was associated with a consistently higher RMST across 5 years of follow-up. The RMST model provides a robust estimation of survival in the presence of proportionality violation and provides estimates for the difference in mean survival times across different time points. HCC surveillance in general was significantly associated with higher RMST, even after adjustment for lead time and multiple confounders. When stratified by etiology of liver disease, HCC surveillance remained associated with higher RMST in people with HBV- or HCV-associated HCC throughout the first 5 years of follow-up compared with those who did not receive HCC surveillance. By contrast, RMST was not significantly different in people with MASLD- or alcohol-associated HCC who received HCC surveillance in the first 3 years of follow-up. Taken together, these findings highlight the association of HCC surveillance with improved survival. The benefits of surveillance appear to be strongest for people with HBV- or HCV-associated HCC but may be more modest for people with MASLD- or alcohol-associated HCC, possibly related to the smaller sample size of the nonviral groups.

A head-to-head prospective study of 54 participants with MASLD cirrhosis who underwent ultrasonography and abbreviated MRI indicated that 35% and 19% of participants had severe limitations to visualization on ultrasonograms and abbreviated MRI scans, respectively.^15^ This study suggested that people with MASLD cirrhosis may have substantial limitations to visualization on both ultrasonographic and cross-sectional imaging, which may impact the detection of suspicious lesions. Data from randomized clinical trials supporting HCC surveillance are lacking.^33^ Meta-analyses of observational studies suggest an association between HCC surveillance and improved survival.^8^ There are very limited observational data examining the association between HCC surveillance and survival, stratified by specific etiologies of liver disease, and the current study helps to fill in this knowledge gap.

### Limitations

The strengths of this study include its large sample size, clinical setting, the inclusion of study centers from West and East Asia, and detailed clinical and laboratory data. However, it is not without limitations. This study was retrospective and, hence, subject to biases associated with all retrospective studies, such as selection bias. Although multivariable RMST analysis was adjusted for multiple demographic and clinical factors, there remains the potential for residual confounding, such as the consumption of alcohol or tobacco.^34^ The sample size in MASLD- and alcohol-associated HCC was smaller compared with that of HBV- and HCV-associated HCC; therefore, the lack of statistical significance in RMST for these etiologies could have been related to sample size, rather than a genuine difference in survival benefit between etiologies. Several participants with MASLD-, HCV-, or alcohol-associated HCC without cirrhosis underwent surveillance despite the absence of guidelines recommending routine surveillance in these situations; however, this is reflective of the heterogeneity in clinical practice.^35^ The cohort lacked representation from Europe, Africa, and the Eastern Mediterranean region; hence, it is unclear whether these findings can be generalized to those regions. There were insufficient data to examine the association of the frequency of surveillance and HCC staging with survival.

## Conclusions

In this cohort study of 1185 participants with HCC, HCC surveillance was associated with improved survival rates for people with HCC in general. The survival benefit of surveillance was consistent across multiple time points in people with HBV- or HCV-associated HCC. The survival benefit of surveillance appeared less consistent for people with MASLD- or alcohol-associated HCC, which may have been related to the relatively modest sample size in the nonviral groups. Prospective studies are required to investigate the association of HCC surveillance with survival outcomes for people with MASLD- and alcohol-associated HCC.

## Notes

Supplement 1. **eMethods 1.** ANCOVA-type regression method for estimating RMST differences **eMethods 2.** Lead time bias adjustment **eFigure.** CONSORT diagram of study participants **eTable 1.** Univariable RMST analysis of people with HCC who underwent surveillance compared to people who did not undergo surveillance **eTable 2.** Univariable RMST analysis of people with HCC who underwent surveillance compared to people who did not undergo surveillance, stratified by etiology **eTable 3.** Univariable RMST analysis of people with HCC who underwent surveillance compared to people who did not undergo surveillance, stratified by etiology, adjusted for lead time with a mean sojourn time of 4 months **eTable 4.** Univariable RMST analysis of people with HCC who underwent surveillance compared to people who did not undergo surveillance, stratified by etiology, adjusted for lead time with a mean sojourn time of 5 months **eTable 5.** Univariable RMST analysis of people with HCC who underwent surveillance compared to people who did not undergo surveillance, stratified by etiology, adjusted for lead time with a mean sojourn time of 6 months **eTable 6.** Multivariable RMST analysis of people with HCC who underwent surveillance compared to people who did not undergo surveillance, excluding people with mixed etiologies of liver disease, stratified by etiology **eTable 7.** Multivariable RMST analysis of people with HCC who underwent surveillance compared to people who did not undergo surveillance, excluding people with less than 6 months of follow-up, stratified by etiology

Supplement 2. **Nonauthor Collaborators.** Liver Cancer Research Network

Supplement 3.
Data Sharing Statement
