# Supplementary material for: Hepatocellular Carcinoma Surveillance and Survival in a Contemporary Asia-Pacific Cohort
Source: JAMA Netw Open. 2025 Jul 11;8(7):e2520294. doi: 10.1001/jamanetworkopen.2025.20294 (PMC12254890; doi:10.1001/jamanetworkopen.2025.20294)
Supplement: Supplement 1. — eMethods 1. ANCOVA-type regression method for estimating RMST differences eMethods 2. Lead time bias adjustment eFigure. CONSORT diagram of study participants eTable 1. Univariable RMST analysis of people with HCC who underwent surveillance compared to people who did not undergo surveillance eTable 2. Univariable RMST analysis of people with HCC who underwent surveillance compared to people who did not undergo surveillance, stratified by etiology eTable 3. Univariable RMST analysis of people with HCC who underwent surveillance compared to people who did not undergo surveillance, stratified by etiology, adjusted for lead time with a mean sojourn time of 4 months eTable 4. Univariable RMST analysis of people with HCC who underwent surveillance compared to people who did not undergo surveillance, stratified by etiology, adjusted for lead time with a mean sojourn time of 5 months eTable 5. Univariable RMST analysis of people with HCC who underwent surveillance compared to people who did not undergo surveillance, stratified by etiology, adjusted for lead time with a mean sojourn time of 6 months eTable 6. Multivariable RMST analysis of people with HCC who underwent surveillance compared to people who did not undergo surveillance, excluding people with mixed etiologies of liver disease, stratified by etiology eTable 7. Multivariable RMST analysis of people with HCC who underwent surveillance compared to people who did not undergo surveillance, excluding people with less than 6 months of follow-up, stratified by etiology [file jamanetwopen-e2520294-s001.pdf]

Supplemental Online Content

Lim RY, Koh B, Ng CH, et al. Hepatocellular carcinoma surveillance and survival in a contemporary Asia-Pacific cohort *JAMA Netw Open*. 2025;8(7):e2520294.
doi:10.1001/jamanetworkopen.2025.20294

eMethods 1. ANCOVA-type regression method for estimating RMST differences

eMethods 2. Lead time bias adjustment

eFigure. CONSORT diagram of study participants

eTable 1. Univariable RMST analysis of people with HCC who underwent surveillance compared to people who did not undergo surveillance

eTable 2. Univariable RMST analysis of people with HCC who underwent surveillance compared to people who did not undergo surveillance, stratified by etiology

eTable 3. Univariable RMST analysis of people with HCC who underwent surveillance compared to people who did not undergo surveillance, stratified by etiology, adjusted for lead time with a mean sojourn time of 4 months

eTable 4. Univariable RMST analysis of people with HCC who underwent surveillance compared to people who did not undergo surveillance, stratified by etiology, adjusted for lead time with a mean sojourn time of 5 months

eTable 5. Univariable RMST analysis of people with HCC who underwent surveillance compared to people who did not undergo surveillance, stratified by etiology, adjusted for lead time with a mean sojourn time of 6 months

eTable 6. Multivariable RMST analysis of people with HCC who underwent surveillance compared to people who did not undergo surveillance, excluding people with mixed etiologies of liver disease, stratified by etiology

eTable 7. Multivariable RMST analysis of people with HCC who underwent surveillance compared to people who did not undergo surveillance, excluding people with less than 6 months of follow-up, stratified by etiology

This supplemental material has been provided by the authors to give readers additional information about their work.

eMethods 1: ANCOVA-type regression method for estimating RMST differences

To estimate the adjusted difference in restricted mean survival time (RMST) between HCC surveillance and no HCC surveillance groups, we applied the ANCOVA-type regression approach proposed by Tian et al.[29] This method models the expected RMST up to a prespecified time horizon τ as a linear function of exposure and baseline covariates, while accounting for right-censoring through inverse probability of censoring weighting.

Let $\mathrm{T_i}$ denote the survival time for subject i, C_i the censoring time, and $Y_i = \min(T_i, \tau)$ the observed follow-up time restricted to τ. The indicator $\widetilde{\Delta_i} = \mathbf{1}(Y_i \leq C_i)$ identifies whether Y_i is uncensored. The covariate vector is defined as $W_i' = (1, Z_i, X_i')$, where: Z_i is a binary exposure indicator (HCC surveillance vs no HCC surveillance), X_i is a vector of baseline covariates, $\beta = (\alpha, \beta_z, \beta_x')$ is the parameter vector. The expected RMST is modeled as: $\mu_\tau(W_i) = \beta' W_i = \alpha + \beta_z Z_i + \beta_x' X_i$. Here, β_z represents the adjusted difference in RMST between the HCC surveillance ($Z = 1$) and the no HCC surveillance ($Z = 0$) groups.

To estimate β, we solve the following estimating equation that weights observations by the inverse of the censoring distribution: $S(\beta) = \frac{1}{n}\sum_{i=1}^{n} \frac{\widetilde{\Delta_i}}{\hat{G}(Y_i)} W_i(Y_i - \beta' W_i) = 0$, where: $\hat{G}(t)$ is the Kaplan-Meier estimator of the censoring distribution, $\frac{\widetilde{\Delta_i}}{\hat{G}(Y_i)}$ are the inverse probability censoring weights, $Y_i - \beta' W_i$ is the residual between observed restricted time and predicted RMST. This estimating equation weights the residuals to account for censoring and minimizes their average across individuals, yielding a consistent estimator $\hat{\beta}$. The asymptotic variance of $\hat{\beta}$ is outlined in the supplementary appendix of Tian et al.

eMethods 2: Lead time bias adjustment

The adjustment of lead time bias is dependent on the sojourn time which is assumed to be exponentially distributed. Sojourn time is defined as the period where the tumour is asymptomatic but detectable via screening. We assumed the mean sojourn time (1/λ) for HCC to be 5 months based on estimations in previous literature and conducted sensitivity analysis using mean sojourn times of 4 and 6 months.[30,31] For patients under surveillance known to be dead at time t, the lead time, E(t), is calculated by using the formula $E(t) = \frac{1-e^{-\lambda t}-\lambda t e^{-\lambda t}}{\lambda(1-e^{-\lambda t})}$. For patients under surveillance known to be alive at time t, the lead time is calculated using the formula $E(t) = \frac{1-e^{-\lambda t}}{\lambda}$. Lead time is corrected by subtracting E(t) from t, the observed survival time.

eFigure: CONSORT diagram of study participants

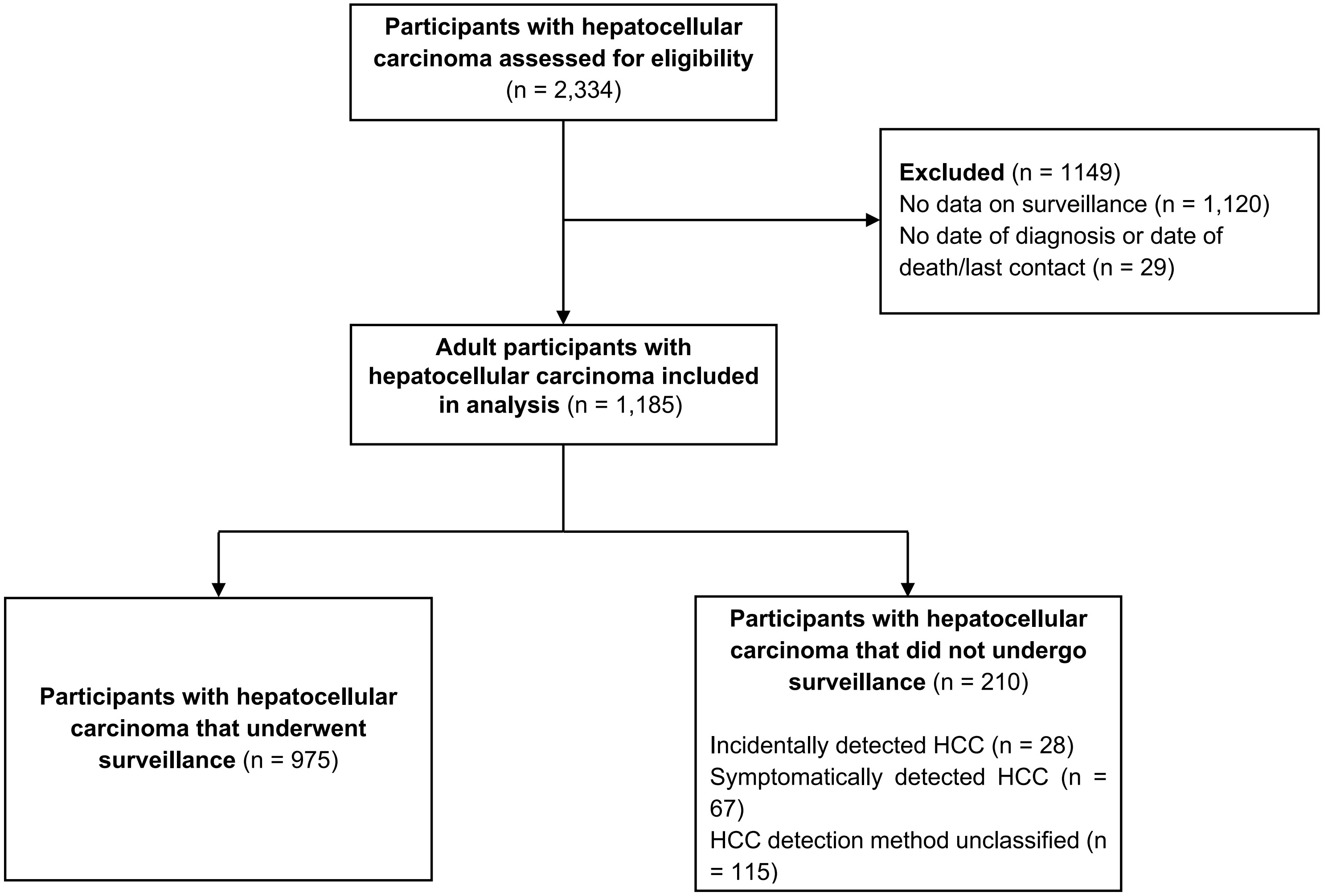

eTable 1: Univariable RMST analysis of people with HCC who underwent surveillance compared to people who did not undergo surveillance

Time, years	RMST (95% CI), years		RMST Difference (95% CI), years	P value
	HCC Surveillance	No HCC Surveillance		
**Crude (Unadjusted)**				
1	0.95 (0.94 - 0.96)	0.82 (0.78 - 0.87)	0.13 (0.08 - 0.17)	<0.001*
2	1.80 (1.77 - 1.83)	1.26 (1.15 - 1.47)	0.34 (0.23 - 0.45)	<0.001*
3	2.58 (2.53 - 2.63)	2.01 (1.84 - 2.18)	0.57 (0.39 - 0.75)	<0.001*
4	3.29 (3.21 - 3.37)	2.46 (2.23 - 2.69)	0.83 (0.58 - 1.08)	<0.001*
5	3.94 (3.84 - 4.05)	2.86 (2.56 - 3.16)	1.08 (0.77 - 1.40)	<0.001*
**Adjusted for lead time, mean sojourn time 4 months**				
1	0.93 (0.91 - 0.94)	0.82 (0.78 - 0.87)	0.10 (0.06 - 0.15)	<0.001*
2	1.75 (1.72 - 1.79)	1.26 (1.15 - 1.47)	0.29 (0.18 - 0.40)	<0.001*
3	2.51 (2.45 - 2.57)	2.01 (1.84 - 2.18)	0.50 (0.32 - 0.68)	<0.001*
4	3.20 (3.11 - 3.28)	2.46 (2.23 - 2.69)	0.74 (0.49 - 0.99)	<0.001*
5	3.83 (3.72 - 3.95)	2.86 (2.56 - 3.16)	0.97 (0.65 - 1.29)	<0.001*
**Adjusted for lead time, mean sojourn time 5 months**				
1	0.92 (0.91 - 0.94)	0.82 (0.78 - 0.87)	0.10 (0.05 - 0.15)	<0.001*
2	1.75 (1.71 - 1.78)	1.26 (1.15 - 1.47)	0.28 (0.17 - 0.39)	<0.001*
3	2.49 (2.43 - 2.55)	2.01 (1.84 - 2.18)	0.49 (0.30 - 0.67)	<0.001*
4	3.18 (3.09 - 3.27)	2.46 (2.23 - 2.69)	0.72 (0.47 - 0.97)	<0.001*
5	3.81 (3.69 - 3.93)	2.86 (2.56 - 3.16)	0.95 (0.63 - 1.27)	<0.001*

**Adjusted for lead time, mean sojourn time 6 months**				
1	0.92 (0.91 - 0.94)	0.82 (0.78 - 0.87)	0.10 (0.05 - 0.15)	<0.001*
2	1.74 (1.70 - 1.77)	1.26 (1.15 - 1.47)	0.27 (0.16 - 0.38)	<0.001*
3	2.48 (2.42 - 2.54)	2.01 (1.84 - 2.18)	0.47 (0.29 - 0.65)	<0.001*
4	3.16 (3.07 - 3.25)	2.46 (2.23 - 2.69)	0.70 (0.45 - 0.95)	<0.001*
5	3.79 (3.67 - 3.91)	2.86 (2.56 - 3.16)	0.93 (0.61 - 1.25)	<0.001*

Legend: *: p<0.05 denotes statistical significance

Abbreviations: RMST, restricted mean survival time; HCC, hepatocellular carcinoma

eTable 2: Univariable RMST analysis of people with HCC who underwent surveillance compared to people who did not undergo surveillance, stratified by etiology

Time, years	RMST (95% CI), years		RMST Difference (95% CI), years	P value
	HCC Surveillance	No HCC Surveillance		
**HBV**				
1	0.96 (0.95 - 0.98)	0.84 (0.76 - 0.91)	0.13 (0.05 - 0.21)	0.001*
2	1.85 (1.81 - 1.90)	1.53 (1.35 - 1.70)	0.33 (0.14 - 0.51)	<0.001*
3	2.68 (2.60 - 2.76)	2.14 (1.86 - 2.42)	0.55 (0.26 - 0.84)	<0.001*
4	3.45 (3.33 - 3.57)	2.68 (2.29 - 3.06)	0.77 (0.37 - 1.18)	<0.001*
5	4.17 (4.01 - 4.33)	3.16 (2.66 - 3.66)	1.01 (0.49 - 1.53)	<0.001*
**HCV**				
1	0.94 (0.92 - 0.96)	0.80 (0.70 - 0.90)	0.15 (0.04 - 0.25)	0.006*
2	1.79 (1.74 - 1.84)	1.40 (1.16 - 1.65)	0.39 (0.14 - 0.64)	0.002*
3	2.54 (2.45 - 2.64)	1.88 (1.49 - 2.26)	0.67 (0.27 - 1.06)	0.001*
4	3.24 (3.10 - 3.37)	2.26 (1.74 - 2.78)	0.98 (0.44 - 1.52)	<0.001*
5	3.86 (3.67 - 4.04)	2.59 (1.93 - 3.25)	1.27 (0.58 - 1.95)	<0.001*
**MASLD**				
1	0.94 (0.91 - 0.97)	0.79 (0.64 - 0.94)	0.15 (0.00 - 0.30)	0.05
2	1.76 (1.68 - 1.85)	1.40 (1.02 - 1.78)	0.36 (-0.03 - 0.75)	0.07
3	2.51 (2.37 - 2.66)	1.91 (1.32 - 2.51)	0.60 (-0.01 - 1.22)	0.06
4	3.19 (2.98 - 3.40)	2.28 (1.50 - 3.06)	0.91 (0.10 - 1.72)	0.03*
5	3.81 (3.53 - 4.09)	2.62 (1.63 - 3.60)	1.19 (0.17 - 2.21)	0.02*
**Alcohol**				
1	0.93 (0.89 - 0.96)	0.86 (0.76 - 0.95)	0.07 (-0.03 - 0.18)	0.18
2	1.74 (1.64 - 1.83)	1.55 (1.31 - 1.78)	0.19 (-0.06 - 0.44)	0.14

3	2.46 (2.29 - 2.62)	2.04 (1.67 - 2.41)	0.42 (0.01 - 0.82)	0.05*
4	3.11 (2.87 - 3.35)	2.39 (1.89 - 2.88)	0.73 (0.18 - 1.28)	0.01*
5	3.72 (3.40 - 4.03)	2.63 (2.02 - 3.24)	1.09 (0.40 - 1.77)	0.002*

Legend: *: $p<0.05$ denotes statistical significance

Abbreviations: RMST, restricted mean survival time; HCC, hepatocellular carcinoma; HBV, hepatitis B virus; HCV, hepatitis C virus; MASLD, metabolic dysfunction-associated steatotic liver disease

eTable 3: Univariable RMST analysis of people with HCC who underwent surveillance compared to people who did not undergo surveillance, stratified by etiology, adjusted for lead time with a mean sojourn time of 4 months

Time, years	RMST (95% CI), years		RMST Difference (95% CI), years	P value
	HCC Surveillance	No HCC Surveillance		
**HBV**				
1	0.95 (0.93 - 0.97)	0.84 (0.76 - 0.91)	0.11 (0.03 - 0.19)	0.006*
2	1.82 (1.76 - 1.87)	1.53 (1.35 - 1.70)	0.29 (0.11 - 0.47)	0.002*
3	2.62 (2.53 - 2.71)	2.14 (1.86 - 2.42)	0.49 (0.19 - 0.78)	0.001*
4	3.37 (3.24 - 3.51)	2.68 (2.29 - 3.06)	0.70 (0.29 - 1.11)	0.001*
5	4.08 (3.90 - 4.26)	3.16 (2.66 - 3.66)	0.92 (0.39 - 1.45)	0.001*
**HCV**				
1	0.92 (0.89 - 0.94)	0.80 (0.70 - 0.90)	0.12 (0.02 - 0.23)	0.02*
2	1.73 (1.67 - 1.79)	1.40 (1.16 - 1.65)	0.33 (0.08 - 0.58)	0.009*
3	2.47 (2.37 - 2.57)	1.88 (1.49 - 2.26)	0.59 (0.19 - 0.99)	0.004*
4	3.14 (2.99 - 3.28)	2.26 (1.74 - 2.78)	0.88 (0.34 - 1.42)	0.001*
5	3.74 (3.54 - 3.93)	2.59 (1.93 - 3.25)	1.15 (0.46 - 1.83)	0.001*
**MASLD**				
1	0.91 (0.87 - 0.95)	0.79 (0.64 - 0.94)	0.12 (-0.03 - 0.28)	0.11
2	1.71 (1.62 - 1.81)	1.40 (1.02 - 1.78)	0.31 (-0.08 - 0.70)	0.12
3	2.43 (2.27 - 2.59)	1.91 (1.32 - 2.51)	0.52 (-0.10 - 1.14)	0.10
4	3.09 (2.86 - 3.32)	2.28 (1.50 - 3.06)	0.81 (0.00 - 1.62)	0.05
5	3.68 (3.38 - 3.98)	2.62 (1.63 - 3.60)	1.06 (0.03 - 2.09)	0.04*
**Alcohol**				
1	0.90 (0.86 - 0.94)	0.86 (0.76 - 0.95)	0.04 (-0.06 - 0.15)	0.43
2	1.68 (1.57 - 1.79)	1.55 (1.31 - 1.78)	0.13 (-0.13 - 0.39)	0.32

3	2.37 (2.19 - 2.55)	2.04 (1.67 - 2.41)	0.33 (-0.08 - 0.75)	0.12
4	3.01 (2.75 - 3.27)	2.39 (1.89 - 2.88)	0.63 (0.07 - 1.18)	0.03*
5	3.60 (3.26 - 3.93)	2.63 (2.02 - 3.24)	0.96 (0.27 - 1.66)	0.007*

Legend: *: p<0.05 denotes statistical significance

Abbreviations: RMST, restricted mean survival time; HCC, hepatocellular carcinoma; HBV, hepatitis B virus; HCV, hepatitis C virus; MASLD, metabolic dysfunction-associated steatotic liver disease

eTable 4: Univariable RMST analysis of people with HCC who underwent surveillance compared to people who did not undergo surveillance, stratified by etiology, adjusted for lead time with a mean sojourn time of 5 months

Time, years	RMST (95% CI), years		RMST Difference (95% CI), years	P value
	HCC Surveillance	No HCC Surveillance		
**HBV**				
1	0.94 (0.92 - 0.96)	0.84 (0.76 - 0.91)	0.11 (0.03 - 0.19)	0.008*
2	1.81 (1.76 - 1.86)	1.53 (1.35 - 1.70)	0.28 (0.10 - 0.46)	0.002*
3	2.61 (2.52 - 2.70)	2.14 (1.86 - 2.42)	0.47 (0.18 - 0.77)	0.002*
4	3.36 (3.23 - 3.49)	2.68 (2.29 - 3.06)	0.68 (0.27 - 1.09)	0.001*
5	4.06 (3.88 - 4.24)	3.16 (2.66 - 3.66)	0.90 (0.37 - 1.43)	0.001*
**HCV**				
1	0.92 (0.89 - 0.94)	0.80 (0.70 - 0.90)	0.12 (0.01 - 0.22)	0.03*
2	1.72 (1.66 - 1.79)	1.40 (1.16 - 1.65)	0.32 (0.07 - 0.57)	0.01*
3	2.45 (2.35 - 2.56)	1.88 (1.49 - 2.26)	0.58 (0.18 - 0.97)	0.005*
4	3.11 (2.96 - 3.26)	2.26 (1.74 - 2.78)	0.86 (0.31 - 1.40)	0.002*
5	3.71 (3.51 - 3.91)	2.59 (1.93 - 3.25)	1.12 (0.43 - 1.81)	0.001*
**MASLD**				
1	0.91 (0.87 - 0.95)	0.79 (0.64 - 0.94)	0.12 (-0.03 - 0.27)	0.13
2	1.70 (1.61 - 1.80)	1.40 (1.02 - 1.78)	0.30 (-0.09 - 0.69)	0.13
3	2.41 (2.25 - 2.58)	1.91 (1.32 - 2.51)	0.50 (-0.12 - 1.12)	0.11
4	3.07 (2.84 - 3.30)	2.28 (1.50 - 3.06)	0.79 (-0.03 - 1.60)	0.06
5	3.65 (3.35 - 3.96)	2.62 (1.63 - 3.60)	1.03 (0.00 - 2.06)	0.05*
**Alcohol**				
1	0.90 (0.85 - 0.94)	0.86 (0.76 - 0.95)	0.04 (-0.07 - 0.15)	0.47
2	1.67 (1.56 - 1.78)	1.55 (1.31 - 1.78)	0.12 (-0.14 - 0.38)	0.36

3	2.36 (2.17 - 2.54)	2.04 (1.67 - 2.41)	0.32 (-0.10 - 0.73)	0.14
4	2.99 (2.73 - 3.25)	2.39 (1.89 - 2.88)	0.60 (0.04 - 1.16)	0.04*
5	3.57 (3.23 - 3.91)	2.63 (2.02 - 3.24)	0.94 (0.24 - 1.64)	0.008*

Legend: *: p<0.05 denotes statistical significance

Abbreviations: RMST, restricted mean survival time; HCC, hepatocellular carcinoma; HBV, hepatitis B virus; HCV, hepatitis C virus; MASLD, metabolic dysfunction-associated steatotic liver disease

eTable 5: Univariable RMST analysis of people with HCC who underwent surveillance compared to people who did not undergo surveillance, stratified by etiology, adjusted for lead time with a mean sojourn time of 6 months

Time, years	RMST (95% CI), years		RMST Difference (95% CI), years	P value
	HCC Surveillance	No HCC Surveillance		
**HBV**				
1	0.94 (0.92 - 0.96)	0.84 (0.76 - 0.91)	0.11 (0.03 - 0.19)	0.009*
2	1.81 (1.75 - 1.86)	1.53 (1.35 - 1.70)	0.28 (0.09 - 0.46)	0.003*
3	2.60 (2.51 - 2.69)	2.14 (1.86 - 2.42)	0.46 (0.17 - 0.76)	0.002*
4	3.34 (3.21 - 3.48)	2.68 (2.29 - 3.06)	0.67 (0.26 - 1.08)	0.001*
5	4.04 (3.86 - 4.22)	3.16 (2.66 - 3.66)	0.88 (0.35 - 1.41)	0.001*
**HCV**				
1	0.91 (0.89 - 0.94)	0.80 (0.70 - 0.90)	0.12 (0.01 - 0.22)	0.03*
2	1.71 (1.65 - 1.78)	1.40 (1.16 - 1.65)	0.31 (0.06 - 0.56)	0.02*
3	2.44 (2.33 - 2.54)	1.88 (1.49 - 2.26)	0.56 (0.16 - 0.96)	0.006*
4	3.09 (2.94 - 3.24)	2.26 (1.74 - 2.78)	0.84 (0.29 - 1.38)	0.003*
5	3.68 (3.49 - 3.88)	2.59 (1.93 - 3.25)	1.09 (0.40 - 1.78)	0.002*
**MASLD**				
1	0.90 (0.87 - 0.94)	0.79 (0.64 - 0.94)	0.12 (-0.04 - 0.27)	0.14
2	1.70 (1.60 - 1.79)	1.40 (1.02 - 1.78)	0.29 (-0.10 - 0.69)	0.14
3	2.40 (2.24 - 2.56)	1.91 (1.32 - 2.51)	0.49 (-0.13 - 1.11)	0.12
4	3.05 (2.81 - 3.28)	2.28 (1.50 - 3.06)	0.77 (-0.05 - 1.59)	0.06
5	3.63 (3.32 - 3.93)	2.62 (1.63 - 3.60)	1.01 (-0.02 - 2.04)	0.06
**Alcohol**				
1	0.89 (0.85 - 0.94)	0.86 (0.76 - 0.95)	0.04 (-0.07 - 0.15)	0.50
2	1.66 (1.55 - 1.77)	1.55 (1.31 - 1.78)	0.11 (-0.15 - 0.37)	0.40

3	2.34 (2.16 - 2.52)	2.04 (1.67 - 2.41)	0.30 (-0.12 - 0.72)	0.16
4	2.97 (2.71 - 3.23)	2.39 (1.89 - 2.88)	0.58 (0.02 - 1.15)	0.04*
5	3.55 (3.20 - 3.89)	2.63 (2.02 - 3.24)	0.92 (0.22 - 1.61)	0.01*

Legend: *: p<0.05 denotes statistical significance

Abbreviations: RMST, restricted mean survival time; HCC, hepatocellular carcinoma; HBV, hepatitis B virus; HCV, hepatitis C virus; MASLD, metabolic dysfunction-associated steatotic liver disease

eTable 6: Multivariable RMST analysis of people with HCC who underwent surveillance compared to people who did not undergo surveillance, excluding people with mixed etiologies of liver disease, stratified by etiology

Etiology	RMST Difference (95% CI, p value), years[a]				
	1 year	2 years	3 years	4 years	5 years
**HBV**					
Lead time unadjusted	0.11 (0.04 - 0.19, p = 0.003)	0.30 (0.12 - 0.47, p = 0.001)	0.50 (0.21 - 0.80, p = 0.001)	0.68 (0.27 - 1.09, p = 0.001)	0.88 (0.35 - 1.42, p = 0.001)
Lead time adjusted					
Mean sojourn time of 4 months	0.10 (0.02 - 0.17, p = 0.01)	0.26 (0.08 - 0.44, p = 0.004)	0.44 (0.15 - 0.74, p = 0.003)	0.61 (0.19 - 1.03, p = 0.004)	0.81 (0.26 - 1.35, p = 0.004)
Mean sojourn time of 5 months	0.09 (0.02 - 0.17, p = 0.02)	0.26 (0.08 - 0.43, p = 0.005)	0.43 (0.13 - 0.73, p = 0.004)	0.59 (0.17 - 1.01, p = 0.006)	0.79 (0.24 - 1.33, p = 0.005)
Mean sojourn time of 6 months	0.09 (0.02 - 0.17, p = 0.02)	0.25 (0.07 - 0.43, p = 0.006)	0.42 (0.12 - 0.71, p = 0.006)	0.58 (0.16 - 1.00, p = 0.007)	0.77 (0.22 - 1.31, p = 0.006)
**HCV**					
Lead time unadjusted	0.10 (-0.02 - 0.21, p = 0.10)	0.36 (0.08 - 0.64, p = 0.01)	0.67 (0.24 - 1.10, p = 0.002)	1.02 (0.44 - 1.59, p = 0.001)	1.25 (0.51 - 2.00, p = 0.001)
Lead time adjusted					
Mean sojourn time of 4 months	0.08 (-0.04 - 0.19, p = 0.21)	0.30 (0.02 - 0.58, p = 0.03)	0.58 (0.15 - 1.01, p = 0.009)	0.89 (0.31 - 1.47, p = 0.003)	1.08 (0.31 - 1.86, p = 0.006)
Mean sojourn time of 5 months	0.07 (-0.05 - 0.19, p = 0.22)	0.29 (0.01 - 0.57, p = 0.04)	0.56 (0.13 - 0.99, p = 0.01)	0.86 (0.28 - 1.45, p = 0.004)	1.04 (0.26 - 1.83, p = 0.009)
Mean sojourn time of 6 months	0.07 (-0.05 - 0.19, p = 0.24)	0.28 (0.00 - 0.56, p = 0.05)	0.54 (0.11 - 0.98, p = 0.01)	0.82 (0.23 - 1.40, p = 0.006)	1.01 (0.23 - 1.79, p = 0.01)
**MASLD**					
Lead time unadjusted	0.17 (-0.01 - 0.34, p = 0.06)	0.47 (0.01 - 0.93, p = 0.05)	0.75 (-0.03 - 1.53, p = 0.06)	1.10 (0.01 - 2.18, p = 0.05)	1.34 (-0.10 - 2.79, p = 0.07)
Lead time adjusted					
Mean sojourn time of 4 months	0.14 (-0.04 - 0.32, p = 0.13)	0.42 (-0.05 - 0.90, p = 0.08)	0.68 (-0.13 - 1.49, p = 0.10)	1.01 (-0.09 - 2.10, p = 0.07)	1.20 (-0.28 - 2.67, p = 0.11)
Mean sojourn time of 5 months	0.13 (-0.05 - 0.31, p = 0.15)	0.42 (-0.05 - 0.89, p = 0.08)	0.67 (-0.14 - 1.48, p = 0.11)	1.01 (-0.11 - 2.12, p = 0.08)	1.17 (-0.31 - 2.64, p = 0.12)

Mean sojourn time of 6 months	0.13 (-0.05 - 0.31, p = 0.16)	0.42 (-0.06 - 0.90, p = 0.08)	0.67 (-0.14 - 1.47, p = 0.11)	0.99 (-0.13 - 2.11, p = 0.08)	1.14 (-0.33 - 2.62, p = 0.13)
**Alcohol**					
Lead time unadjusted	0.07 (-0.06 - 0.20, p = 0.28)	0.28 (-0.02 - 0.59, p = 0.07)	0.62 (0.11 - 1.13, p = 0.02)	1.06 (0.38 - 1.73, p = 0.002)	1.55 (0.71 - 2.40, p < 0.001)
Lead time adjusted					
Mean sojourn time of 4 months	0.06 (-0.07 - 0.19, p = 0.36)	0.24 (-0.08 - 0.55, p = 0.143)	0.54 (0.02 - 1.07, p = 0.043)	0.94 (0.25 - 1.63, p = 0.008)	1.40 (0.53 - 2.27, p = 0.002)
Mean sojourn time of 5 months	0.06 (-0.07 - 0.19, p = 0.38)	0.23 (-0.09 - 0.54, p = 0.16)	0.52 (-0.01 - 1.05, p = 0.05)	0.91 (0.22 - 1.61, p = 0.01)	1.37 (0.49 - 2.24, p = 0.002)
Mean sojourn time of 6 months	0.06 (-0.08 - 0.19, p = 0.39)	0.21 (-0.11 - 0.53, p = 0.19)	0.51 (-0.02 - 1.04, p = 0.06)	0.89 (0.19 - 1.60, p = 0.01)	1.34 (0.46 - 2.22, p = 0.003)

Abbreviations: RMST, restricted mean survival time; HCC, hepatocellular carcinoma; HBV, hepatitis B virus; HCV, hepatitis C virus; MASLD, metabolic dysfunction-associated steatotic liver disease

[a] Adjusted for sex, age, ethnicity, type 2 diabetes mellitus, body mass index and Child-Pugh Turcotte Class

eTable 7: Multivariable RMST analysis of people with HCC who underwent surveillance compared to people who did not undergo surveillance, excluding people with less than 6 months of follow-up, stratified by etiology

Etiology	RMST Difference (95% CI, p value), years[a]				
	**1 year**	**2 years**	**3 years**	**4 years**	**5 years**
**HBV**					
Lead time unadjusted	-0.01 (-0.01 - 0.00, p = 0.14)	0.05 (-0.04 - 0.14, p = 0.30)	0.14 (-0.06 - 0.35, p = 0.17)	0.24 (-0.08 - 0.56, p = 0.14)	0.36 (-0.09 - 0.81, p = 0.12)
Lead time adjusted					
Mean sojourn time of 4 months	-0.02 (-0.03 - -0.01, p = 0.003)	0.02 (-0.08 - 0.12, p = 0.72)	0.08 (-0.12 - 0.29, p = 0.43)	0.17 (-0.16 - 0.49, p = 0.32)	0.27 (-0.19 - 0.73, p = 0.25)
Mean sojourn time of 5 months	-0.02 (-0.03 - -0.01, p = 0.001)	0.01 (-0.09 - 0.11, p = 0.82)	0.07 (-0.14 - 0.28, p = 0.51)	0.15 (-0.18 - 0.47, p = 0.38)	0.25 (-0.21 - 0.71, p = 0.29)
Mean sojourn time of 6 months	-0.02 (-0.04 - -0.01, p = 0.001)	0.01 (-0.09 - 0.10, p = 0.91)	0.06 (-0.15 - 0.26, p = 0.60)	0.13 (-0.20 - 0.46, p = 0.43)	0.23 (-0.23 - 0.69, p = 0.33)
**HCV**					
Lead time unadjusted	0.04 (-0.01 - 0.10, p = 0.11)	0.20 (0.00 - 0.40, p = 0.05)	0.40 (0.04 - 0.75, p = 0.03)	0.62 (0.10 - 1.13, p = 0.02)	0.78 (0.10 - 1.45, p = 0.02)
Lead time adjusted					
Mean sojourn time of 4 months	0.03 (-0.03 - 0.08, p = 0.36)	0.15 (-0.05 - 0.35, p = 0.15)	0.31 (-0.05 - 0.67, p = 0.09)	0.51 (-0.01 - 1.02, p = 0.06)	0.62 (-0.07 - 1.30, p = 0.08)
Mean sojourn time of 5 months	0.02 (-0.03 - 0.08, p = 0.42)	0.14 (-0.06 - 0.34, p = 0.18)	0.29 (-0.07 - 0.65, p = 0.11)	0.48 (-0.04 - 1.00, p = 0.07)	0.58 (-0.11 - 1.27, p = 0.10)
Mean sojourn time of 6 months	0.02 (-0.04 - 0.08, p = 0.47)	0.13 (-0.08 - 0.33, p = 0.22)	0.28 (-0.08 - 0.64, p = 0.13)	0.44 (-0.08 - 0.96, p = 0.10)	0.55 (-0.14 - 1.24, p = 0.12)
**MASLD**					
Lead time unadjusted	0.04 (-0.03 - 0.11, p = 0.22)	0.13 (-0.19 - 0.45, p = 0.44)	0.27 (-0.32 - 0.85, p = 0.37)	0.56 (-0.32 - 1.43, p = 0.21)	0.78 (-0.51 - 2.06, p = 0.24)
Lead time adjusted					
Mean sojourn time of 4 months	0.02 (-0.05 - 0.10, p = 0.53)	0.09 (-0.25 - 0.42, p = 0.62)	0.21 (-0.41 - 0.83, p = 0.51)	0.48 (-0.42 - 1.37, p = 0.29)	0.66 (-0.66 - 1.98, p = 0.32)
Mean sojourn time of 5 months	0.02 (-0.06 - 0.10, p = 0.60)	0.08 (-0.25 - 0.42, p = 0.64)	0.20 (-0.42 - 0.83, p = 0.52)	0.48 (-0.45 - 1.40, p = 0.31)	0.64 (-0.69 - 1.96, p = 0.35)

Mean sojourn time of 6 months	0.02 (-0.06 - 0.10, p = 0.65)	0.08 (-0.26 - 0.42, p = 0.64)	0.20 (-0.42 - 0.83, p = 0.52)	0.46 (-0.48 - 1.40, p = 0.34)	0.61 (-0.72 - 1.94, p = 0.37)
**Alcohol**					
Lead time unadjusted	0.00 (-0.02 - 0.03, p = 0.76)	0.06 (-0.08 - 0.19, p = 0.44)	0.22 (-0.09 - 0.54, p = 0.17)	0.52 (0.03 - 1.00, p = 0.04)	0.87 (0.19 - 1.55, p = 0.01)
Lead time adjusted					
Mean sojourn time of 4 months	-0.01 (-0.04 - 0.03, p = 0.58)	0.01 (-0.15 - 0.16, p = 0.94)	0.15 (-0.18 - 0.48, p = 0.37)	0.42 (-0.08 - 0.92, p = 0.10)	0.74 (0.04 - 1.43, p = 0.04)
Mean sojourn time of 5 months	-0.01 (-0.05 - 0.02, p = 0.51)	0.00 (-0.16 - 0.15, p = 0.96)	0.13 (-0.19 - 0.46, p = 0.42)	0.40 (-0.10 - 0.90, p = 0.12)	0.71 (0.01 - 1.41, p = 0.05)
Mean sojourn time of 6 months	-0.01 (-0.05 - 0.02, p = 0.45)	-0.02 (-0.17 - 0.14, p = 0.82)	0.12 (-0.21 - 0.45, p = 0.48)	0.38 (-0.13 - 0.89, p = 0.14)	0.68 (-0.03 - 1.39, p = 0.06)

Abbreviations: RMST, restricted mean survival time; HCC, hepatocellular carcinoma; HBV, hepatitis B virus; HCV, hepatitis C virus; MASLD, metabolic dysfunction-associated steatotic liver disease

[a] Adjusted for sex, age, ethnicity, type 2 diabetes mellitus, body mass index and Child-Pugh Turcotte Class
