# Supplementary material for: Hepatocellular Carcinoma Surveillance and Survival in a Contemporary Asia-Pacific Cohort
Source: JAMA Netw Open. 2025 Jul 11;8(7):e2520294. doi: 10.1001/jamanetworkopen.2025.20294 (PMC12254890; doi:10.1001/jamanetworkopen.2025.20294)
Supplement: Supplement 2. — Nonauthor Collaborators. Liver Cancer Research Network [file jamanetwopen-e2520294-s002.pdf]

Supplemental Online Content: Nonauthor Collaborators
*First name, last name, and suffix (if applicable) are required and will appear in PubMed.

***Group Name(s): Liver Cancer Research Network**							
***First Name and Middle Initial(s)**	***Last Name**	***Suffix (eg, Jr, III)**	Academic Degrees	Institution	Location (city, state/province, country)	Role or Contribution, eg, chair, principal investigator	Group (if more than 1 Group listed in the byline) and/or Subgroup (eg, Steering Committee)
Padaki Nagaraja	Rao			Department of Hepatology, Asian Institute of Gastroenterology, Hyderabad, India			
Mithun	Sharma			Department of Hepatology, Asian Institute of Gastroenterology, Hyderabad, India			
Duvvur Nageshwar	Reddy			Department of Hepatology, Asian Institute of Gastroenterology, Hyderabad, India			
Charlotte	Kench			A.W. Morrow Gastroenterology and Liver Centre, Australian Liver Transplant Unit, Royal Prince Alfred Hospital, Sydney, New South Wales, Australia			
Shirin	Salimi			A.W. Morrow Gastroenterology and Liver Centre, Australian Liver Transplant Unit, Royal Prince Alfred Hospital, Sydney, New South Wales, Australia			
Abdul-Hamid	Sabih			A.W. Morrow Gastroenterology and Liver Centre, Australian Liver Transplant Unit, Royal Prince Alfred Hospital, Sydney, New South Wales, Australia			
Majd B.	Aboona			University of Arizona College of Medicine-Phoenix, Phoenix, Arizona, USA			
Claire S.	Faulkner			University of Arizona College of Medicine-Phoenix, Phoenix, Arizona, USA			
Pooja	Rangan			University of Arizona College of Medicine-Phoenix, Phoenix, Arizona, USA			
Nicholas L	Syn			Yong Loo Lin School of Medicine, National University of Singapore, Singapore			
Margaret LP	Teng			Yong Loo Lin School of Medicine, National University of Singapore, Singapore Division of Gastroenterology and Hepatology, Department of Medicine, National University Hospital, Singapore, Singapore			
Maureen	Da Costa			Division of Hepatobiliary and Pancreatic Surgery, Department of Surgery, National University Hospital, Singapore, Singapore			
Hae Lim	Lee			Division of Hepatology, Department of Internal Medicine, Catholic University of Korea, Seoul, Republic of Korea			
Ming-Hua	Zheng			Department of Hepatobiliary Surgery, The First Affiliated Hospital of Wenzhou Medical University, Wenzhou, Zhejiang, China			
Chong-Ming	Zheng			Department of Hepatobiliary Surgery, The First Affiliated Hospital of Wenzhou Medical University, Wenzhou, Zhejiang, China			
Masahito	Nakano			Division of Gastroenterology, Department of Medicine, Kurume University School of Medicine, Kurume, Japan			
Toru	Nakamura			Division of Gastroenterology, Department of Medicine, Kurume University School of Medicine, Kurume, Japan			
Keisuke	Amano			Division of Gastroenterology, Department of Medicine, Kurume University School of Medicine, Kurume, Japan			
Takuya	Kuwashiro			Division of Metabolism and Endocrinology, Department of Medicine, Faculty of Medicine, Saga University, Saga, Japan			
