# Supplementary material for: Hepatocellular Carcinoma Surveillance and Survival in a Contemporary Asia-Pacific Cohort
Source: JAMA Netw Open. 2025 Jul 11;8(7):e2520294. doi: 10.1001/jamanetworkopen.2025.20294 (PMC12254890; doi:10.1001/jamanetworkopen.2025.20294)
Supplement: Supplement 3. — Data Sharing Statement [file jamanetwopen-e2520294-s003.pdf]

Data Sharing Statement

Lim. Hepatocellular Carcinoma Surveillance and Survival in a Contemporary Asia-Pacific Cohort. *JAMA Netw Open*. Published July 11, 2025.
doi:10.1001/jamanetworkopen.2025.20294

Data
Data available: Yes
Data types: Deidentified participant data
How to access data: Data is available from the authors upon request
When available: With publication

Supporting Documents
Document types: None

Additional Information
Who can access the data: After approval
Types of analyses: After approval
Mechanisms of data availability: After approval
